# Polypharmacology of anthelmintics at host and parasite ion channels

**DOI:** 10.1371/journal.ppat.1013977

**Published:** 2026-02-20

**Authors:** John D. Chan, Spencer S. Ericksen, Mostafa Zamanian

**Affiliations:** 1 Global Health Institute, University of Wisconsin-Madison, Madison, Wisconsin, United States of America; 2 Department of Pathobiological Sciences, University of Wisconsin-Madison, Madison, Wisconsin, United States of America; 3 UW Carbone Cancer Center, University of Wisconsin-Madison, Madison, Wisconsin, United States of America; McGill University, CANADA

## Introduction

An ideal anti-infective drug specifically, or at least selectively, engages a pathogen target. However, compared to viruses or bacteria, the genomes and proteomes of eukaryotic pathogens closely resemble those of their hosts. Many anti-infective chemotherapies have polypharmacology at both host and pathogen targets. Several drugs targeting human receptors have their origins as anti-parasitic drugs. For example, the dewormer phenothiazine gave rise to anti-histamine and anti-psychotic drug classes, and anti-protozoal quinazolines led to the development of quaalude tranquilizers. Anti-schistosomal chemotherapies are another illustration of this polypharmacology, and classes of compounds that kill schistosomes are derived from chemistry initially developed against host targets. While host receptor activity complicates lead development, it offers an advantage by leveraging a broader literature to deconvolute drug mechanisms.

### 1. How were the anti-schistosomal compounds praziquantel and meclonazepam discovered?

**Fig 1:**
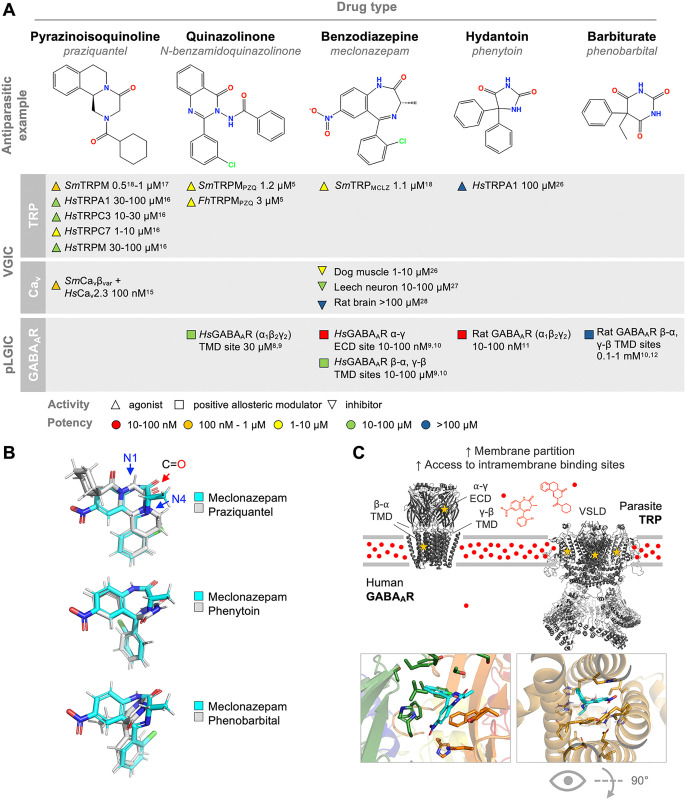
Anthelmintic pharmacophores have activity against both GABA_A_Rs and VGICs. **A.** Numerous structurally similar compounds with anti-schistosomal activity have actions on voltage-gated ion channel family members (e.g., Transient Receptor Potential (TRP) channels or voltage-gated Ca^2+^ (Ca_v_) channels) and GABAergic pentameric ligand-gated ion channels (GABA_A_Rs). Selected examples of these interactions from literature (reference number in superscript) and range of experimental drug concentration used in the assay are shown. △ = agonist, □ = positive allosteric modulator, ▽ = inhibitor, with fill color reflecting potency. *Hs* = human. *Sm* = *Schistosoma mansoni*. *Fh* = *Fasciola hepatica*. **B.** Small molecule alignment (BCL::MolAlign [50]) for meclonazepam (cyan) and several ligands from **(A)** (gray). 3D conformers show similar positioning of ring systems and heteroatoms; N1, N4 (blue arrows), and carbonyl group (red arrow) are annotated. **C.** Top—Drug binding sites for GABA_A_Rs (α1β2γ2 receptor structure PDB 6X3X [10]) and TRPs (no solved schistosome structure has been published, a homology model of schistosome Smp_333650 with *Drosophila* channel 5VKQ is shown). Visible sites given the channel orientation are starred. Drug binding sites include the GABA_A_R ECD between α and γ subunits, GABA_A_R TMD between β and α units (1/2 sites visible), GABA_A_R TMD between γ and β subunits (not starred, site positioned towards rear view of channel), and TRP VSLD (3/4 sites visible, one positioned in rear view of channel). Red symbols indicate partitioning of compounds into the plasma membrane. Bottom—Predicted binding pose of meclonazepam within the human GABA_A_R (left, ECD site between green γ2 and orange α1 subunits) and schistosome TRP VSLD (right) binding site [30]. Note, the schistosome TRP site is portrayed rotated 90 degrees, from above the channel, to provide a view unobstructed by helices S1–S4.

Schistosomiasis is a disease caused by infection with blood-dwelling parasitic flatworms. The current therapy, praziquantel, was discovered by Bayer/Merck and an experimental lead, meclonazepam, was discovered by Hoffmann-La Roche. Both originated by screening sedative libraries against parasites [1,2]. The rationale for why these libraries were chosen is not documented, but the connection between anti-parasitics and sedatives is not entirely arbitrary. Quaaludes have their origin in quinazoline anti-parasitic drugs [3]. Barbiturates also cause worms to release from the mesenteric vasculature and shift to the portal vein and liver. The anti-epilepsy drug and barbiturate derivative phenytoin has *in vivo* anti-schistosomal activity in mice [4]. The classes of compounds listed above are structurally similar. The core heterocyclic structures of meclonazepam (benzodiazepine), praziquantel (pyrazinoisoquinoline), and the experimental derivative N-benzamidoquinazolinone, BZQ (quinazoline, [5]), share a coupled ring with two nitrogens and a carbonyl group. These groups are also found in the ureic structure of barbiturates and phenytoin. Aligning these molecules’ 3D conformers highlights this similarity; note the similar placement of nitrogens, carbonyl groups, and ring systems in Fig 1B.

### 2. Why do sedatives and related molecules have anthelmintic activity?

Several compounds in Fig 1A have sedative effects on humans. They also have anti-parasitic effects on schistosomes. Unfortunately, in the example of meclonazepam, these two effects manifest at the same dosing range [6]. Praziquantel, although derived from a weakly sedating chemical series [7], does not display dose-limiting sedation. Both compounds cause schistosome paralysis and tegument damage. In the case of praziquantel, this triggers detection by the host immune system and parasite clearance. However, the molecular targets that underpin these effects were unknown until recently.

Various classes of compounds with sedative effects act through GABA_A_Rs [8–12], which are pentameric Ligand Gated Ion Channels (pLGICs) that mediate the flow of chloride ions. Schistosome genomes lack GABA_A_R orthologs, and so sedative and anti-parasitic outcomes are not mediated by the same receptors [13]. Compounds in Fig 1A are privileged chemical scaffolds known to bind multiple biological targets. Some of the earliest work on praziquantel and meclonazepam speculated that they likely act directly on a mechanism transporting inorganic ions such as Ca^2+^ to cause parasite muscle contraction [14]. Voltage-gated Ca^2+^ (Ca_v_) channels were initially hypothesized as targets. Co-expression of a schistosome accessory Ca_v_β subunit sensitizes a *mammalian* Ca_v_ɑ channel to praziquantel [15]. But it was never directly shown that praziquantel could cause Ca^2+^ influx through a *flatworm* Ca_v_ɑ channel. Rather, praziquantel and meclonazepam were later found to activate different members of the Voltage-Gated Ion Channel (VGIC) family, Transient Receptor Potential (TRP) channels [16–19]. These effects occur at concentrations that mirror the activity on worms *in vitro* (hundreds of nanomolar for praziquantel, and low micromolar for meclonazepam).

How do the concentrations of compounds needed to achieve effects at targets in Fig 1 compare to levels achieved in human dosing? In the case of praziquantel, the EC_50_ at the TRP target is below the concentration encountered by worms *in vivo*. The *C*_max_ for the active R-enantiomer of praziquantel in pediatric school-age children is 1 µM [20], and levels of the R-trans-4-OH-PZQ metabolite (~10 µM) are also within the active range of this compound on the parasite TRP channel [21]. In the case of meclonazepam, a 1 mg dose has been shown to reach a *C*_max_ of ~30 nM [22]. Assuming linear scaling, a ~20 mg anti-schistosomal dose (assuming 70 kg body weight, [6]) would have a *C*_max_ of ~600 nM in systemic circulation. While this is below the concentration of meclonazepam that activates a schistosome TRP channel (~1 µM) and kills worms *in vitro* (~3 µM), schistosomes reside in the mesenteric vasculature, prior to first pass metabolism, and are likely exposed to concentrations of praziquantel and benzodiazepines as much as 10× higher than the systemic *C*_max_ [23,24].

While GABA_A_Rs and VGICs are distinct ion channel classes, there is precedent for polypharmacology at these targets. Many compounds known to target GABA_A_Rs also modulate VGICs—both parasite and mammalian TRPs [5,16–18,25], as well as Ca_v_ channels [15,26–28]. GABA_A_Rs and VGICs are the focus of our discussion, given their relevance to anti-parasitic activity and host side effects, but it is noted these are not the only targets engaged by compounds in Fig 1. For example, binding and functional assays profiling other targets engaged by praziquantel and a meclonazepam derivative have been published [29,30].

### 3. Why would GABAergic sedatives act on parasite TRP channels?

Compounds such as the benzodiazepine meclonazepam target both GABA_A_Rs and TRPs, but act on different regions of these channels (Fig 1C). Benzodiazepines bind GABA_A_Rs at the extracellular interface of α-γ subunits and the interface of the transmembrane domains (TMDs) of β-α and γ-β subunits [31]. But benzodiazepines are predicted to bind parasite TRP channels within the voltage sensor-like domain (VSLD) [18], an intra-membrane region within each subunit that regulates channel gating. The predicted benzodiazepine binding sites of GABA_A_Rs and TRPs do not have obvious sequence or structural similarity. If the ligand binding sites of these two channel types are different, what explains their shared pharmacology? We can propose several possibilities.

First, recent GABA_A_R and TRP structures show that drug binding sites are also occupied by lipids (reviewed in [32]). Lipids in the benzodiazepine GABA_A_R α-γ and α-β binding sites are displaced by drug [10]. Many lipid TRP agonists that bind near or within the VSLD have also been reported. Examples include phosphatidylinositol 4,5-bisphosphate, C3 [33], pregnenolone sulfate [34], testosterone [35], arachidonic acid, and fatty acids [36]. Solved structures for schistosome TRPs complexed with anthelmintics will be needed to determine whether these VSLD lipid binding sites are conserved in flatworm channels and if they overlap with anthelmintic binding sites. However, if similar lipids are involved in regulation of both GABA_A_R and TRP channels, this may explain polypharmacology across these classes.

Second, both the GABA_A_R TMD and TRP VSLD binding sites are situated within the plasma membrane and are accessible to compounds that partition into the lipid bilayer, in contrast to extracellular or cytosolic sites, which are accessible to aqueous-solubilized drugs [37]. Sedatives and anesthetics are known to intercalate into the plasma membrane, and their activity at TRP channels may relate to physicochemical properties that promote drug partitioning at high concentrations near the VSLD [38].

### 4. How does this inform future anti-schistosomal drug discovery?

Having identified the parasite targets of praziquantel and meclonazepam, we can now ask mechanistic questions regarding their pharmacological modulation and biological function. Schistosome TRPs have been expressed as homomeric channels *in vitro*, but we do not know whether subunits form homomeric or heteromeric assemblies *in vivo*. If different subunits interact, and each were targeted by unique ligands, might an admixture of compounds have even greater anti-parasitic effects? For example, heteromeric voltage-gated potassium (K_v_) channels can be composed of subunits with distinct agonist binding sites, and co-administration of compounds has synergistic effects [39]. This approach may even address key limitations of praziquantel, such as lack of efficacy against juvenile, liver-stage worms.

Will all TRP agonists display anti-schistosomal activity? Chemotypes may vary in magnitude and duration of evoked Ca^2+^ influx, corresponding to differences in anti-parasitic efficacy. And as TRP channels are known multimodal sensors, it is also important to consider whether *in vitro* assay conditions match *in vivo* conditions. For example, the chemical probe FPL-64176 is an agonist at the target of meclonazepam [18], but only causes paralysis of worms *in vitro* when worms are shifted from 37 °C to cooler temperatures (23 °C) [40]. This mirrors studies on TRP channels that show cooling potentiates chemical-evoked currents [41], or reciprocally that chemical treatment sensitizes cooling-evoked currents [42].

Possibly, variation in the ability of different stimuli to activate the channel is relevant to their normal biological function. Praziquantel and meclonazepam cause a massive, persistent, and non-desensitizing Ca^2+^ influx. But endogenous ligands may act on schistosome TRPs more subtly. For example, in other organisms TRP channels have sensory functions (taste sensing by human TRPM5 [36], *Brugia malayi* migration towards host-associated cues through OSM-9 [43]).

Finally, helminth TRPs may have unique features not found in mammalian orthologs. The schistosome benzodiazepine target, Smp_333650, clusters near the broader TRPM subfamily, although it is unusual in featuring N-terminal ankyrin domains. Along with other invertebrate TRPM-like channels (e.g., nematode ‘TRPS’ ced-11 [44]), these divergent clades merit further exploration to determine if they have atypical properties and pharmacology.

### 5. Broader relevance to anti-parasitic drug development

Several points raised above have parallels in chemotherapy of other parasites. Praziquantel and meclonazepam are inactive on *Fasciola* liver flukes, but target-based screens on TRPs are capable of identifying broad-spectrum ligands [5]. There is also precedent for roundworm anthelmintics that target TRPs and pLGICs. Diethylcarbamazine was discovered in the 1940s, as a derivative of the anthelmintic piperazine. While piperazine modulates GABA_A_Rs, diethylcarbamazine is proposed to activate *Brugia* TRP channels (e.g., TRP-2, [45]). Ivermectin’s mechanism shares some similarities with benzodiazepines and barbiturates discussed above, but it acts on different pLGICs, glutamate-gated chloride channels (GluCls). The ivermectin binding site overlaps with a phospholipid (1-Palmitoyl-2-oleoyl-sn-glycero-3-phosphoserine, POPS) binding site at the interface of two glc-1 TMDs [46]. Lipids often allosterically regulate channel activity, and both benzodiazepines and macrocyclic lactones bind similar lipid-occupied sites and display positive allosteric modulator (PAM) activity at their respective targets. It is unknown if the ivermectin binding site on other sensitive GluCls also binds lipids or what role these may play in channel regulation.

As lipid interactions with ion channels become better understood, lipid-derived small molecules could also serve as leads for drug development. POPS binding at *C. elegans* glc-1 increases affinity of the channel for glutamate [46], and there is precedent for lipids as anti-parasitics. For instance, miltefosine activates trypanosome ion channels [47], while other alkylphospholipid analogs demonstrate *in vivo* efficacy against schistosomes [48].

## Conclusions

Many anthelmintics were historically identified in phenotypic screens agnostic to drug targets, but were later found to act on ion channels. In the case of anti-schistosomal drugs, tranquilizer-like compounds were found to kill parasites—though via targets distinct from those causing human sedation. Why some compounds display polypharmacology at GABAergic and VGIC targets remains an open question. However, similarities in intra-membrane ligand binding sites may offer a partial explanation. Indeed, shortly after praziquantel’s discovery, similarities were noted with the membrane-intercalating properties of anesthetics and tranquilizers [49]. More work is needed to understand gating mechanisms of these TRP channels, but allosteric sites such as the VSLD may present opportunities for parasite-selective chemotherapies. Although few new chemotypes have entered the anthelmintic pipeline, robust TRP functional assays and structural biology advances suggest these targets will yield promising leads.
